# The Potential of a High Protein-Low Carbohydrate Diet to Preserve Intrahepatic Triglyceride Content in Healthy Humans

**DOI:** 10.1371/journal.pone.0109617

**Published:** 2014-10-16

**Authors:** Eveline A. Martens, Blandine Gatta-Cherifi, Hanne K. Gonnissen, Margriet S. Westerterp-Plantenga

**Affiliations:** Department of Human Biology, Maastricht University, Maastricht, The Netherlands; The Chinese University of Hong Kong, Hong Kong

## Abstract

**Background:**

Protein supplementation has been shown to reduce the increases in intrahepatic triglyceride (IHTG) content induced by acute hypercaloric high-fat and high-fructose diets in humans.

**Objective:**

To assess the effect of a 12-wk iso-energetic high protein-low carbohydrate (HPLC) diet compared with an iso-energetic high carbohydrate-low protein (HCLP) diet on IHTG content in healthy non-obese subjects, at a constant body weight.

**Design:**

Seven men and nine women [mean ± SD age: 24±5 y; BMI: 22.9±2.1 kg/m^2^] were randomly allocated to a HPLC [30/35/35% of energy (En%) from protein/carbohydrate/fat] or a HCLP (5/60/35 En%) diet by stratification on sex, age and BMI. Dietary guidelines were prescribed based on individual daily energy requirements. IHTG content was measured by ^1^H-magnetic resonance spectroscopy before and after the dietary intervention.

**Results:**

IHTG content changed in different directions with the HPLC (CH_2_H_2_O: 0.23±0.17 to 0.20±0.10; IHTG%: 0.25±0.20% to 0.22±0.11%) compared with the HCLP diet (CH_2_H_2_O: 0.34±0.20 vs. 0.38±0.21; IHTG%: 0.38±0.22% vs. 0.43±0.24%), which resulted in a lower IHTG content in the HPLC compared with the HCLP diet group after 12 weeks, which almost reached statistical significance (*P* = 0.055).

**Conclusions:**

A HPLC vs. a HCLP diet has the potential to preserve vs. enlarge IHTG content in healthy non-obese subjects at a constant body weight.

**Trial Registration:**

Clinicaltrials.gov NCT01551238

## Introduction

Energy-restricted high-protein diets are commonly applied as interventions aiming to induce body weight loss and related improvements in metabolic profile [1], [2]. On the other hand, high carbohydrate-high fat diets may increase the susceptibility for overeating, which may result in the development of metabolic disturbances [3], [4], [5]. Visceral adipose tissue (VAT) volume has been linked to the metabolic disturbances associated with obesity, such as a diminished insulin sensitivity and dyslipidemia [6]. However, high ectopic lipid content, especially intrahepatic triglyceride (IHTG) content, and not VAT volume, is an independent risk factor for these metabolic disturbances [7], [8], [9]. In obese subjects, increases in BMI, total adipose tissue volume, or VAT volume were only associated with increases in insulin resistance and disturbances in very-low-density lipoprotein (VLDL)-TG metabolism when IHTG content was increased simultaneously [9]. Furthermore, insulin sensitivity was lower and VLDL secretion was higher in obese subjects with a high IHTG content (>5.5% of liver volume), matched on VAT volume [8]. No differences were observed between subjects with a high VAT volume (>1 100 cm^3^) but similar IHTG content [8]. Therefore, measuring IHTG content as a marker of metabolic function in dietary intervention studies is highly relevant.

Several human studies suggest that high protein intake may modulate IHTG content. A 4-wk uncontrolled study showed that protein supplementation was associated with a reduction in IHTG content in obese subjects [10]. In two short-term dietary intervention studies, protein supplementation reduced the increases in IHTG content induced by hypercaloric high-fat and high-fructose diets in healthy subjects [11], [12]. However, the longer-term effects of dietary protein content on IHTG content, thereby excluding the possible effects of metabolic disturbances and changes in body weight are unknown. Therefore, in the context of prevention of metabolic disturbances, the aim of this dietary intervention study was to assess the effect of a 12-wk iso-energetic high protein-low carbohydrate (HPLC) diet compared with an iso-energetic high carbohydrate-low protein (HCLP) diet on IHTG content in healthy non-obese subjects, at a constant body weight.

## Subjects and Methods

The Medical Ethical Committee of Maastricht University approved the study, and all subjects gave written informed consent. The study was registered on clinicaltrials.gov with Identifier: NCT01551238. The protocol for this trial and supporting CONSORT checklist are available as supporting information; see Checklist S1 and protocol S1.

### Study subjects

Based on the study by Thetaz et al. [12], power analysis showed that with an α of 0.05 and a β of 0.80, at least 7 subjects per group were needed to show a difference in IHTG content between the dietary interventions of this study.

**Figure 1:**
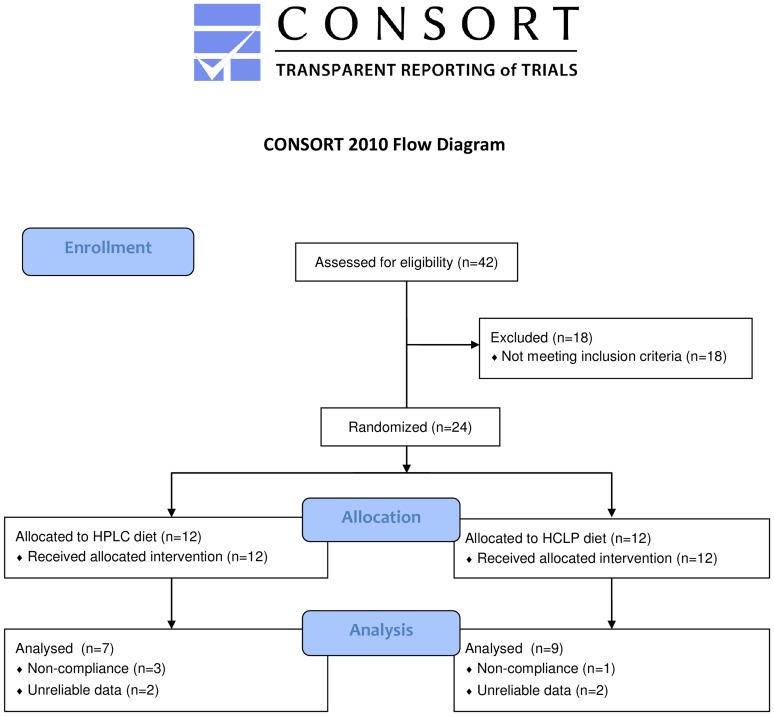
Flow diagram (CONSORT). HCLP, high-carbohydrate low-protein; HPLC, high-protein low-carbohydrate.

Twenty-four subjects were recruited by advertisements in local newspapers and on notice boards at the university. Data from measurements of four subjects were unreliable, and were therefore excluded from analysis. Four subjects had to be excluded from the data analysis because of non-compliance with the designated protein intake, as shown by the urinary nitrogen biomarker. The baseline characteristics of the excluded subjects did not differ from the total sample. Overall, 16 subjects (7 men and 9 women) were included in the final data analysis (**Figure 1**).

Subjects underwent a screening that included anthropometric measurements and the completion of questionnaires eliciting information about health, smoking behaviour, use of medications, and alcohol consumption. Subjects were aged between 19 and 31 y had a BMI between 19.0 and 25.3 kg/m^2^. Age and BMI were equally divided between males and females. Subjects were non-smoking, not using more than moderate amounts of alcohol (>10 drinks/wk), were weight stable (body weight change <3 kg during the last 6 mo and had no planned weight change during the study period), and were not using medication or supplements except for oral contraceptives in women.

The validated Dutch translation of the Baecke Activity Questionnaire was used to measure habitual physical activity scores, which were converted into a standardized individual physical activity level [13], [14].

### Dietary intervention

The study used a single-blind parallel design in which subjects were randomly allocated to the HPLC [30/35/35% of energy (En%) from protein/carbohydrate/fat] or the HCLP (5/60/35 En%) diet following stratification on sex, age and BMI. Detailed dietary guidelines were prescribed based on individual daily energy requirements, calculated as the basal metabolic rate determined with the formula of Harris and Benedict [15] times the physical activity level [16]. This was carried out in order to keep body weight constant. Moreover, subjects were told that they were expected to maintain their body weight and physical activity level. The dietary guidelines included a variety of recipes for breakfasts, lunches, dinners and snacks, consisting of commercially available food items and the amounts that had to be consumed to reach the intended diet compositions. The guidelines included the prescription to consume additional protein [HPLC: whey with α-lactalbumin (Hiprotal Whey Protein Alpha, FrieslandCampina Domo EMEA, Amersfoort, The Netherlands)] or carbohydrate [HCLP: maltodextrin (Fantomalt, Nutricia, Zoetermeer, The Netherlands)] by shakes twice daily (**Table 1**).

**Table 1:** Sample menu for the HPLC and HCLP diets.

	HPLC	HCLP
**Breakfast**	Low-fat yoghurt	Whole-grain juice with forest fruits
	Breakfast cereals	Breakfast cereals
	Kiwi	Apple
	Semi-skimmed milk	Orange juice
**Lunch**	Whole-wheat bread	Whole-wheat bread
	Margarine	Margarine
	Cheese spread	Apple spread
	Chicken breast	Hazelnut paste
**Snack**	Protein shake	Carbohydrate shake
**Dinner**	White rice	White rice
	French beans	French beans
	Chicken breast	Red cabbage with apple
	Walnuts	Red pepper
	Olive oil	Olive oil
	Savoury sauce	Savoury sauce
**Snack**	Protein shake	Carbohydrate shake
	Cereal bar	Corn crisps
	Apple	Pear

The amounts that were prescribed to consume were based on individual daily energy requirements.

### Body weight and biomarker of protein intake

At baseline and at weeks 5, 9 and 12, body weight was measured by using a digital balance in order to check whether it remained constant, and 24-h urinary nitrogen excretion was determined as biomarker of protein intake throughout the intervention period.

### IHTG content

MRI and proton magnetic resonance spectroscopy (^1^H-MRS) measurements were performed using a 1.5-T whole-body scanner (Intera; Philips Healthcare, Best, The Netherlands). Sagittal, coronal and transversal images through the right lobe in the lower third of the liver were obtained by MRI (slice thickness 8 mm; repetition time/echo time, 500/15 ms; matrix 180×96) in order to gain anatomical and positional information of the liver, ^1^H-MRS enabled to measure IHTG content by distinguishing hepatic water from a spectrum of IHTG. Fatty acid chains that represent different proton moiety were separated by the relative magnitude of resonance [17]. A 20×20×20 mm volume of interest was positioned, avoiding the lateral margin of the liver parenchyma, major blood vessels, and intrahepatic bile ducts. The position of the volume of interest was the same at the baseline measurement and at the measurement after the intervention. Following shimming (10×10×10 cm volume), eight spectra were acquired using a Q-body coil [18]. Point-resolved spectroscopy sequence (PRESS) was used for spatial localization and signal acquisition using a protocol set up by Schrauwen-Hinderling et al (repetition time/echo time, 4000/33 ms; number of signals averaged fat/water, 64/16) [19]. Water signal was suppressed using 150 Hz frequency-selective prepulses. The unsuppressed water signal was measured in the same volume of interest under the same shimming conditions, and was used as a reference signal [19]. Spectra were fitted using the Java-based magnetic resonance user interface (jMRUI) package fitted in the domain using a non-linear least-squares algorithm (AMARES) [19], [20], [21]. Areas of resonance from protons of methylene [-(CH_2_)_n_-] and methyl (CH_3_) in the fatty acid chains, relative to the area of protons of H_2_O, were used as a measure for the IHTG content. A more extensive description of the applied post processing procedure has been published previously [18], [19], [21], [22], [23].

### Statistical analysis

All statistical analyses were performed by using SPSS version 20 for Macintosh OS X (SPSS Inc.). Differences in subject characteristics and anthropometrics between the diet groups were assessed using Factorial ANOVA. Mann-Whitney tests were used to test for differences in IHTG content between the diet groups.

Factorial ANOVAs with repeated measures were used to test whether nitrogen excretion, body weight, and BMI changed over time within the diet groups, and to test whether these effects differed between the diet groups. Data on IHTG content were log transformed before these statistical analyses were performed. Differences were regarded statistically significant if *P*<0.05. Data are presented as mean ± SD.

## Results

### Subject characteristics

Subject characteristics did not differ between the diet groups at baseline (**Table 2**). No adverse events were reported.

**Table 2:** Subject characteristics, anthropometrics and IHTG content in the HPLC and HCLP diet groups at baseline and after 12 weeks.

	Baseline		After 12 weeks	
	HPLC	HCLP	HPLC	HCLP
**No. of subjects (M/F)**	7 (4/3)	9 (3/6)		
**Age (y)**	23±5	25±5		
**Baecke total score**	9.4±1.0	8.9±1.1		
**Height (cm)**	173±8	170±9		
**BW (kg)**	67.6±6.6	64.5±8.2	67.8±6.2	64.3±7.5
**BMI (kg/m^2^)**	22.6±2.0	22.3±2.1	22.7±2.3	22.2±2.0
**IHTG content**				
**CH_2_H_2_O**	0.23±0.17	0.34±0.20	0.20±0.10*	0.38±0.21*
**IHTG (%)**	0.25±0.20	0.38±0.22	0.22±0.11*	0.43±0.24*

Values represent numbers or means ± SD.

*Trend (*P* = 0.055) for factorial ANOVA with repeated measures between diet groups.

BW, body weight; HCLP, high-carbohydrate low-protein; HPLC, high-protein low-carbohydrate; IHTG, intrahepatic triglyceride.

### Body weight and biomarker of protein intake

The condition of stable body weight was met in both arms. Baseline nitrogen excretion did not differ significantly between the HPLC (12.2±3.6 g/d) and HCLP (11.4±3.4 g/d) diets. Nitrogen excretion increased significantly in the HPLC (23.3±4.4 g/d, *P*<0.05), and decreased in the HCLP (6.5±1.3 g/d, *P* = 0.001) diet group compared with baseline. Significant differences in protein intake between the diet groups were confirmed in that nitrogen excretion differed significantly between the diet groups throughout the intervention period (*P* = 0.001).

### IHTG content

IHTG content decreased with the HPLC (CH_2_H_2_O: 0.23±0.17 to 0.20±0.10; IHTG%: 0.25±0.20% to 0.22±0.11%) and increased with the HCLP diet (CH_2_H_2_O: 0.34±0.20 vs. 0.38±0.21; IHTG%: 0.38±0.22% vs. 0.43±0.24%), although not reaching statistical significance (Table 2). These changes of IHTG content in different directions resulted in a lower IHTG content in the HPLC compared with the HCLP diet group after 12 weeks, which almost reached statistical significance (*P* = 0.055).

## Discussion

Protein supplementation has been shown to reduce the increases in IHTG content induced by acute hypercaloric high-fat and high-fructose diets in humans. In the context of prevention of metabolic disturbances, we assessed the longer-term effects of iso-energetic diets with large contrasts in relative protein content on IHTG content in healthy non-obese subjects, thereby excluding the possible effects of changes in body weight. The present study showed that IHTG content changed in different directions with the HPLC compared with the HCLP diet, which resulted in a lower IHTG content in the HPLC compared with the HCLP diet group after 12 weeks, which almost reached statistical significance. The observation that the condition of stable body weight was met in both diet groups confirmed that this observation was not affected by changes in body weight. The successful implementation of the dietary intervention in each diet group was confirmed by urinary nitrogen concentrations.

A proposed mechanism for the modulation of IHTG content by a high protein intake is the stimulation of hepatic lipid oxidation due to the high energetic demand for amino acid catabolism and ketogenesis [24], [25]. Hepatic lipid oxidation may also be stimulated by an increased bile acid production in response to high protein intake, a process that may also inhibit lipogenesis [26]. Furthermore, protein-induced glucagon secretion inhibits de novo lipogenesis and stimulates hepatic ketogenesis [27], [28].

On the other hand, previous studies showed that hyper- and eucaloric high-carbohydrate diets stimulated the synthesis of fatty acids from dietary glucose via de novo lipogenesis [29], [30], [31], [32]. The carbohydrate-induced de novo lipogenesis correlated with plasma TG concentrations [30], [31]. Increased IHTG deposition was shown to be the result of hepatic TG-synthesis exceeding the rate of its secretion into VLDL [29], [30]. The relationship between high carbohydrate intake and increased VLDL-TG concentrations, possibly caused by stimulation of VLDL-TG production and inhibition of VLDL-TG clearance [33], may result in increases in hepatic TG concentrations, and subsequently in IHTG content [31]. Protein supplementation has been observed to blunt the increase in IHTG content induced by short-term hypercaloric high-fructose intake in healthy subjects [12]. Therefore, it is likely that the observed trend for a difference in IHTG content after the consumption of the HPLC compared with the HCLP diet may be the result of combined effects involving changes in protein and carbohydrate intake.

The strength of the present study was the application of an iso-energetic macronutrient intervention for a period that was long enough to reliably measure changes in IHTG content, thereby excluding the possible effects of metabolic disturbances and changes in body weight. Our healthy, non-obese subjects had a low IHTG content at baseline, which relates to a healthy metabolic function. This may exhibit a strong resistance to metabolic changes in response to dietary interventions when stimuli such as a high-fat or a high-fructose intake are absent [11], [12]. This indicates that high protein-low carbohydrate diets may be biologically less relevant for inducing a significant decrease in IHTG content in healthy subjects. Nevertheless, the observations of this study are relevant by suggesting that high protein-low carbohydrate diets may be beneficial for the prevention of metabolic disturbances in healthy subjects. Measurements of energy expenditure, body composition, body fat distribution including ectopic fat deposition, and metabolic function are needed to assess the translation of high-protein diets as a treatment strategy into a preventive measure. By addressing ectopic fat deposition in response to dietary interventions, this study adds to the discussion of the clinical relevance of dietary protein for the prevention of obesity. It would also be relevant to implement the measurement of IHTG content in studies assessing the effects of high-protein diets during body weight loss and weight maintenance in patients with metabolic disturbances.

To conclude, a HPLC vs. a HCLP diet has the potential to preserve vs. enlarge IHTG content in healthy non-obese subjects at a constant body weight.

## Supporting Information

Checklist S1 **CONSORT Checklist.**
(DOC)

Protocol S1 **Study Protocol.**
(PDF)
